# Cultural Adaptation and Psychometric Testing of the Short Form of Iranian Childbirth Self Efficacy Inventory

**DOI:** 10.5812/ircmj.11741

**Published:** 2013-11-05

**Authors:** Mahboubeh Khorsandi, Mohammad Asghari Jafarabadi, Farzaneh Jahani, Mohammad Rafiei

**Affiliations:** 1 Arak University of Medical sciences, Arak, IR Iran; 2 Medical Education Research Center, Faculty of Health, Tabriz University of Medical Sciences, Tabriz, IR Iran

**Keywords:** Childbirth, Self-Efficacy, Pregnancy, Psychometric Testing

## Abstract

**Background:**

To assess maternal confidence in her ability to cope with labor, a measure of childbirth self efficacy is necessary.

**Objectives:**

This paper aims to assess the cultural adaptation and psychometric testing of the short form of childbirth self-efficacy Inventory among Iranian pregnant women.

**Patients and Methods:**

In this descriptive-methodological study, we investigated 383 Iranian pregnant women in the third trimester. They were recruited from the outpatient prenatal care clinic of Taleghani Hospital and an urban health center from August to November 2011. Content validity was evaluated by a panel of specialists after adding two religious items. The women completed the inventory and the demographic characteristics questionnaire in an interview room. The internal consistency and construct validity were assessed by Cronbach’s alpha and by exploratory and confirmatory factor analyses, respectively. Known group analysis on gravity assessed the discriminant validity of the measure.

**Results:**

Content validity of the short form of the Iranian childbirth self-efficacy Inventory was confirmed. Factor analyses supported the conceptual two-factor structure of measure and hence supported its construct validity. The internal consistency was approved for the total scale and both subscales. The instrument differentiated prim gravid from multigravida women in the total scale and the efficacy expectancy subscale.

**Conclusions:**

Validity and reliability of the measure supports the use of the short form of the instrument as a clinical and research instrument in measuring childbirth self-efficacy among Iranian pregnant women.

## 1. Background

The quality of the perception of childbirth and coping with it, as a developmental event, affects a woman’s self-efficacy (1, 2). A woman’s confidence in her ability plays a major role in coping (3), and self-efficacy towards that is inversely associated with the level of labor pain perception (4). During labor, self-efficacy expectancy would assist a woman to reflect her capabilities in coping with this stressful situation and to perform required behaviors (2).

To investigate maternal confidence in coping with labor, Lowe (1993) developed the childbirth self-efficacy inventory (CBSEI) to: 1) promote the conceptual development of delivery confidence and 2) help with effective nursing interventions, (2). Since its development, Outcome Expectancy (OE) and Efficacy Expectancy (EE) have been evaluated by CBSEI in many studies. With a reasonable level of validity and reliability (2, 3, 5-9), the original CBSEI included 62 items, loading in a four factor structure and two repetitive sets of items (10).

However, pregnant women’s confidence in their coping behavior showed no differences between the first and second stage of labor in the previous studies (6, 11). Since it is too hard to find different responses between two stages of labor based on repetitive and parallel sets of items in pregnant women, it is not recommended to use it for both assessments and there are concerns in this regard. Ip et al. (2008) developed a short-form Chinese CBSEI by deleting two repetitive subscales (OE-15 and EE-15) to solve the problem of the repetitive and lengthy structure of the original childbirth self-efficacy inventory. The short form consists of two parallel OE-16 and EE-16 subscales, containing the same 16 items for evaluating childbirth coping behavior (7). The short form CBSEI has adequate predictive validity, construct (both convergence and discriminant) validity, internal consistency and test-retest reliability (7, 10). In addition, a study by Khorsandi et al. (2008), they suggested to add religious items to the short form of CBSEI to adapt with the Iranian culture (8), which was considered in this study. Compared to the lengthy structure of the original CBSEI, the short form is that it takes a short time to complete and has not been used in Iranian women. To our knowledge, no such instrument has been used, if any, in Iranian population.

## 2. Objectives

This study was designed to test the cultural compatibility and psychometric properties (including validity and reliably) of the modified short form of Iranian Childbirth Self-Efficacy Inventory (ICBSEI-36) in Iranian pregnant women. The following specific aims were addressed in this study:

1. To determine the adaption of ICBSEI-36 with the added religious items.

2. To determine the factor model for the short form of ICBSEI-36.

3. To determine the discriminability of the responses obtained from the factor structure of the ICBSEI-36 between prim gravid and multigravida women.

4. To determine the internal consistency of factor structure of the ICBSEI-36.

## 3. Patients and Methods

### 3.1. Participants

In this methodology methodological-cross sectional study, a convenience sample of 383 Iranian pregnant women in their third trimester of pregnancy who were visited at the outpatient prenatal care clinic of Taleghani Hospital and an urban health center for prenatal visit of Arak City, Iran, for prenatal visit was recruited from August to November 2011. The inclusion criteria included being in the third trimester of pregnancy and being able to read Persian. In addition, multiparous women with a history of previous cesarean section were excluded from the study, because Iranian obstetricians follow the rule of ‘once a cesarean, always a cesarean’.

Five or more participants per item or a total sample size of two hundred has been reported to be adequate for factor analysis (12, 13). In this study, a total of 383 Iranian pregnant women were recruited which was an adequate sample size.

### 3.2. Instruments

The original CBSEI consists of 62 items and four subscales with two repetitive sets of items; each one is completed during the first and second stage of labor. Each of the four subscales consists of the same 15 items addressing common behaviors to cope with childbirth. Also, was added one item, “focus on person helping me in labor’’, to OE-16 and EE-16 subscales (10).

When compared to the lengthy structure of the original CBSEI with two sets of repetitive items, the short form of the CBSEI has OE-16 and EE-16 parallel subscales (7), consisting of the same 16 items to measure perceived self-efficacy in coping with the whole labor process. The coping items of the short-form CBSEI are scored on a 10-point self-report scale ranging from one (not at all helpful) to ten (very helpful) for the OE-16 subscale, and from one (not at all sure) to ten (very sure) for the EE-16 subscale. For each subscale, the score is computed by adding the scores of the items; the total score ranges between 16 and 160 with higher scores indicating higher levels of OE or EE for labor (10). To make the questionnaire compatible with the Iranian culture, two religious items were added. Therefore, the adopted measure (ICBSEI-36) had eighteen items and each subscale yielded a scale score between 18 and 180.

### 3.3. Methodology

The Persian translation of the inventory was carried out in a forward–backward translation procedure. The tool was translated by a midwife in forward translation. Afterwards, two local professional translators with experience in living in English-speaking countries translated it back to US English. Some revisions were made in the Persian translation after comparing the US English back-translation and the original version. Thirty women completed the questionnaire. Then, we made some corrections in wording in this stage to remove possible linguistic problems.

An expert panel of fifteen professional in obstetrics and gynecology, health education, nursing and midwifery assessed the CBSEI qualitatively for content validity. In this process, a few items were revised and finally, to be compatible with the Iranian culture, two religious items were added.

A questionnaire with background characteristics was also completed including age (years), parity (primiparous and multiparous), educational level (illiterate, junior high school education, high school diploma, university education), husband’s educational level ((illiterate, junior high school education, high school diploma, university education), occupation (housewife, employed in governmental sector, self-employed), husband’s occupation (employed in governmental sector, self-employed, unemployed), antenatal class attendance (yes or no), Insurance type (social insurance, health care, other), household income (100-299, 300-399 and = > 400 thousand Toman monthly).

### 3.4. Ethical Considerations

Permission to use the original CBSEI was obtained from the author. The ethical committee of Arak University of Medical Sciences approved the study (Ethical code: 89-83-10 and date of approval: 2011/4/9). The participants were informed of the general nature of the study and were assured of the confidentiality of the data. Written informed consent was obtained from all participants.

### 3.5. Statistical Analysis

The data were summarized as mean (SD). Univariate normality of data was examined by skewness and kurtosis, according to which absolute values greater than three (14) and absolute values more than ten (15) are extreme. Ceiling and floor effects were considered present if more than fifteen percent of the respondents had the lowest or highest possible score, respectively (16). Internal consistency reliability was investigated by Cronbach’s Alpha and Guttman’s Split half reliability. Values higher than 0.7 were considered satisfactory (17). Exploratory Factor Analysis (EFA) was used to examine the underlying relationships between observed variables. EFA solutions were extracted by Principal Axis Factoring, utilizing Varimax Rotation Method with Kaiser Normalization. The Scree plot was used to determine the optimal number of factors (17). KMO (Kaiser-Meyer-Olkin) measure of sampling adequacy and Bartlett's test of sphericity were used for the evaluation of model adequacy. KMO indicates the proportion of variance in variables explained by underlying factors. High values (>0.7) indicate model adequacy for data. P- Values < 0.05 in Bartlett's test of sphericity indicate the usefulness of the model for data. The important relationships between items and factors were determined based on factor loading values of 0.3 or higher (18). Confirmatory Factor Analysis (CFA) was conducted with weighted least squares estimation method, asymptomatic covariance matrix weight matrix and covariance matrix as input to assess how well the EFA extracted model fitted the observed data. Fit indices and their acceptable values used in the analysis were χ2 / df < 5, Root Mean Square Error of Approximation (RMSEA) < 0.08 and Standardized Root Mean Square Residual (SRMR) < 0.1 and also, Comparative Fit Index (CFI), Normed Fit Index (NFI), Non-Normed Fit Index (NNFI) >0.90. Independent samples t and Hotelling T square tests were performed to determine the differences between primigravid and multigravid women (which were theoretically expected to differ (2, 7, 19, 20)) in OE and EE subscales and also in the total score. Statistical analysis was performed using SPSS 13 (SPSS Inc., Chicago, IL) and LISREL 8.80 (Scientific Software International Inc., 2007). P-values less than 0.05 were considered significant.

## 4. Results

### 4.1. Sample Characteristics

Out of 400 study cases, 383 returned the questionnaire (Response rate = 95.75%). Of them, 68.73% were primiparous (n = 255) and the rest were multiparous (n =128). They were all married and the majority (42.6 %) had high school diploma. Most of the respondents did not attend childbirth education classes (83 %). The Mean maternal age was 32.8 (SD 7.26) years and the mean gestational age was 29.4 weeks (SD 9.4). Mean maternal weight and height were 71.01 (SD 11.7) kg and 158.62 (SD 19.9) cm, respectively (also for other characteristics see Table 1). 

**Table 1.** Background Characteristics of study participants (n=383) ^a ^

Characteristics	No.(%)
Parity	
Primiparous	255 (66.6)
Multiparous	128 (33.4)
**Educational level**	
illiterate	11 (2.9)
Junior high school education	158 (41.5)
High school diploma	163 (42.8)
University education	49 (12.9)
**Husband’s Educational level**	
illiterate	16 (4.2)
Junior high school education	176 (46.7)
High school diploma	146 (38.7)
University education	39 (10.3)
**Occupation**	
Housewife	356 (93.4)
Employed in Governmental sector	13 (3.4)
Self-employed	12 (3.1)
**Husband’s Occupation**	
Employed in Governmental sector	51 (17.0)
Self-employed	236 (78.7)
Unemployed	13 (4.3)
**Antenatal class attendance**	
Yes	318 (84.8)
No	57 (15.2)
**Insurance Type**	
social security	168 (48.1)
Health care	129 (37.0)
Other	52 (14.9)
**Household income (monthly)**	
100-299 thousand Toman	140 (42.0)
300-399 thousand Toman	162 (48.6)
= > 400 thousand Toman	31 (9.3)

^a^ If the sum of frequency does not match the total number of 383, there are non-responses in those characteristics.

The normality of each observed variable based on skewness and kurtosis (due to large sample sizes) was confirmed (absolute skewness < 3 and absolute kurtosis measure< 10) (Table 2). 

**Table 2.** Summary of statistics of CBSEI-C336 Subscale Scores (n = 383) ^a ^

	Mean	Std. Deviation	Skewness	Kurtosis
**OE**	134.55	32.06	-0.86	0.70
**EE**	112.07	37.11	-0.13	-0.66
**Total**	246.62	62.85	-0.49	0.17

^a^ The possible range of OE and EE score is 18-180 and for total score is 36-360

### 4.2. Feasibility

Ceiling effects were detected for 26 persons (6.8%) in the OE subscale and for 12 persons (3.1%) in the EE subscale. There was no Floor effect for theses subscales.

### 4.3. Content Validity

In qualitative evaluation of the measure, experts provided written feedback on the clarity and relevancy of the content of the ICBSEI-36 items to the Iranian culture and the content validity of the measure was generally supported. It is noteworthy that some items were revised based on the qualitative suggestions of the panel experts.

### 4.4. Reliability

The Cronbach’s alpha was 0.92 for the total scale and 0.88 and 0.88 for the subscales measuring OE and EE respectively, indicating adequate internal consistency (>0.7). Guttman's split half index for total scale (0.78), OE subscale (0.82) and EE subscale (0.83) showed a satisfactory (>0.7) split half reliability.

### 4.5. Construct Validity

For evaluating construct validity, both EFA and CFA were performed for the items of OE and EE subscales.

#### 4.5.1. EFA

In this analysis, KMO measures of sampling adequacy were 0.902 and 0.903 for OE and EE respectively, which supported the sampling adequacy of the data for modeling for these subscales. Bartlett’s test of sphericity gave P < 0.05. The Scree plot for both subscales supported the uni-dimensionality of each one.

Cut-off values ≥ 0.3 for factor loadings showed that all items related to OE and EE subscales were reasonably loaded on theses subscales (Table 3). The results suggested that each ICBSEI-36 subscale was one-dimensional. 

**Table 3.** Factor Loadings for OE and EE Subscales

Items of OE	Factor Loadings	Items of EE	Factor Loadings
**9. Stay on top of each contraction**	0.653	4. Keep myself in control	0.715
**8. Concentrate on thinking about the baby**	0.642	9. Stay on top of each contraction	0.712
**10. Think positively**	0.634	10. Think positively	0.690
**12. Tell myself that I can do it**	0.628	2. Get ready for each contraction	0.686
**16. Focus on the person helping me in labour**	0.624	12. Tell myself that I can do it	0.684
**15. Listen to encouragement from the person helping me**	0.614	7. Keep myself calm	0.669
**7. Keep myself calm**	0.567	5. Think about relaxing	0.641
**5. Think about relaxing **	0.552	11. Not think about the pain	0.581
**14. Concentrate on getting through one contraction at a time**	0.528	6. Concentrate on an object in the room to distract myself	0.570
**3. Use breathing during labour contractions**	0.517	8. Concentrate on thinking about the baby	0.567
**17. I Praise God and ask for help from him**	0.514	3. Use breathing during labour contractions	0.563
**11. Not think about the pain**	0.504	1. Relax my body	0.561
**2. Get ready for each contraction**	0.492	13. Think about others in my family	0.558
**1. Relax my body**	0.488	16. Focus on the person helping me in labour	0.539
**13. Think about others in my family**	0.465	14. Concentrate on getting through one contraction at a time	0.538
**6. Concentrate on an object in the room to distract myself**	0.460	18. Walking between labour pain	0.444
**4. Keep myself in control**	0.407	17. I Praise God and ask for help from him	0.350
**18. Walking between labour pain**	0.399	15. Listen to encouragement from the person helping me	0.312

#### 4.5.2. CFA

The model showed reasonably good fit indices (x2/degrees of freedom(df) = 3.70<5; SRMR = 0.07 < 0.1, RMSEA = 0.000 <0.08 ((90% confidence interval (CI)) = (0.000; 0.010)), CFI= 0.99 > 0.90, NFI= 1.00 > 0.90, NNFI = 1.01 > 0.90 and good support for the two-factor structure of the ICBSEI-36 (MacCallum, Browne, & Sugawara, 1996; Kline, 2005; Marsh, Balla, & Hau, 1996).

**Figure 1.**
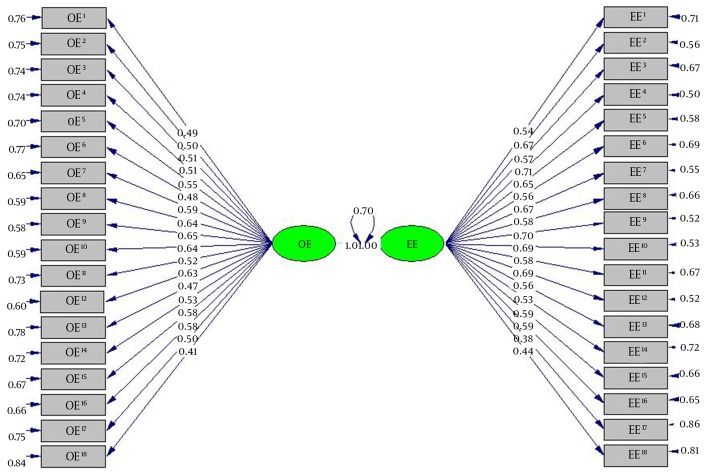
CFA Factor Loadings for OE and EE Subscales All factor loadings were statistically significant (All P<0.05) and there was a significant correlation between OE and EE subscales (P < 0.05).

Based on this model, factor loadings indicate significant loadings on the two-factor solution (Figure 1). Standardized factor loadings ranged from 0.41 to 0.64 on the OE subscale, and from 0.38 to 0.71 on the EE subscale, with all items demonstrating moderate to strong factor loadings (above 0.30) ( 21 ) (Figure 1). 

The statistical significance of the two-factor correlations (r = 0.70, P < .01) supported the hypothesis that the two factors (OE and EE subscales) were highly related dimensions of childbirth self-efficacy.

### 4.6. Convergent Validity

The correlation coefficient between the subscales and the total scale was 0.89 (P < 0.01) for the OE subscale and 0.92 (P < .01) for the EE subscale, and the correlation coefficient between the subscales was 0.65 (P < .01), indicating a significant overlap between the two subscales.

### 4.7. Known Group Analysis

To assess the discriminant validity of the scales, the results of multivariate test for comparing multigravid and primigraid women showed a significant simultaneous difference (Hotteling T2 = 0.025, F (2,368) = 4.64 and P = 0.010). Also, univariate analysis showed a significant difference between primi an multigravid women in the total scale (t = -2.118, df = 369 and P = 0.035) and the EE subscale (t = -2.811, df = 369 and P = 0.005) with a higher mean score for multigravid women (Table 4), providing evidence supportive for the discriminant (construct) validity of the ICBSEI-36 as indicated by known group procedure. However, for the OE subscale, the result was insignificant (t = -0.884, df = 369 and P = 0.377). 

**Table 4.** Results for comparison between multigravid and primigraid women (n = 371) ^a ^

PMGravid	No	Mean	Std. Deviation	t^b^	df	Sig. (2-tailed)
**OE**						
Primigravid	255	134.54	32.40	-0.88	369	0.377
Multigravid	116	137.66	29.69			
**EE**						
Primigravid	255	109.00	35.83	-2.81	369	0.005
Multigravid	116	120.50	38.00			
**Total**						
Primigravid	255	243.54	60.66	-2.12	369	0.035
Multigravid	116	258.16	63.83			

^a^ The possible range of OE and EE score is 18-180 and for total score is 36-360.

^b^ T-test base on equal variances assumed (Homogeneity of variance using Levene's test confirmed the equality of variance test for OE, EE and total scores (all P > 0.05).

## 5. Discussion

Findings provided support for acceptable reliability and validity of ICBSEI-36 for the assessment of childbirth self-efficacy among pregnant women in Iran. In a study conducted in a sample of the Chinese population in Hong Kong, the measure showed a reasonable level of validity and reliability as a self-report measure of women’s childbirth self-efficacy (7, 10).

### 5.1. Feasibility

Ceiling effects of 6.8% and 3.1% were detected for OE and EE subscales respectively but there was no floor effect for theses subscales, which confirmed the feasibility of the measure in the Iranian population. No studies related to this instrument present measure of ceiling and floor effects.

### 5.2. Content Validity

The content validity of ICBSEI-36 was supported based on the evaluation of a panel of experts. The same procedure was performed and the same results were achieved in a study conducted by Gallo et al. (2011) (10).

### 5.3. Reliability

The ICBSEI-36 had acceptable internal consistency (Cronbach’s alpha fulfilling the criteria), indicating a satisfactory degree of consistency among items for each subscale. In other studies, high internal consistency reliability has been reported for the original measure (0.82–0.96) (2, 5, 6) and also for the short form of the measure (7, 10).

### 5.4. Construct Validity

The results of EFA and CFA provided evidence the uni-dmentionality structure for each subscale of the ICBSEI-I36, reflecting the consistency of the two dimensions of OE and EE subscales with the original factor structure identified in previous researches (7). The results of other studies also suggest that each ICBSEI-I36 subscale is one-dimensional, leading in a two-factor structure (2, 5, 6, 10).

### 5.5. Convergent Validity

High values of the correlation between subscales, which indicated a significant overlap between the two subscales, supported the convergent validity of the measure. Similarly, the convergent validity with the Chinese self-efficacy scale was reflected by a moderate correlation for the two subscales (7).

### 5.6. Discriminant Validity

The parity differences observed for the ICBSEIC-36, with higher subscale and scale scores for multigravid versus primigravid women in EE, were consistent with the theoretical construct of the measure (19). According to Bandura (1997), direct experiencing of any event, such as childbirth, affects the perceptions of efficacy beliefs, as a powerful source of information (19). Women with positive previous experiences in labor are more probable to have a higher perceived self-efficacy for a forthcoming birth and to report a positive childbirth experience (22). This results were in line with those reported by Ip et al. (2008) and Lowe (1993) in which EE scores differentiated primigravid women from multigravida women (2, 7). However, Gao et al (2011) observed higher scores of both OE-16 and EE-16 subscales in multigravid than primigravid women (10); hence, further research is needed to explore the discriminant validity of the OE scale.

The strength of this study includes a large sample size, fulfilling the requirement of 5 or more participants per item for factor analysis. However, most samples were well educated, married, and from middle-class society; thus, generalizability of the results is limited. In addition, it is recommended to perform CFA in a different sample.

This study showed that the ICBSEI-36 was a valid, reliable and culturally compatible measure which could be used as a research instrument. Also, it was found to be short and feasible enough to be used as a clinical instrument for measuring childbirth self-efficacy in Iranian pregnant women. It could be employed as a measure to perform educational interventions in women who need to improve their confidence in coping ability for labor. In addition, due to its feasibility and time conserving nature, this short form could be used by midwives, nurses and clinicians who are involved in the care of pregnant mothers in order to identify mothers who need psychological support.
